# Salivary Oxidative Stress and Antioxidant Markers in Oral Leukoplakia: A Systematic Review and Meta-Analysis

**DOI:** 10.3390/antiox15020218

**Published:** 2026-02-06

**Authors:** Patryk Wiśniewski, Magdalena Sulewska, Zuzanna Rybaczek, Kornelia Szymańska, Julia Nowakowska, Marcel Chrobot, Maja Podedworna, Karolina Doroszczyk, Paulina Murtaś, Małgorzata Pietruska

**Affiliations:** 1 Department of Periodontal and Oral Mucosa Diseases, Medical University of Białystok, ul. Waszyngtona 13, 15-269 Białystok, Poland; magdalena.sulewska@umb.edu.pl (M.S.); malgorzata.pietruska@umb.edu.pl (M.P.); 2 Students’ Research Group on Oral Mucosal Diseases, Department of Periodontal and Oral Mucosa Diseases, Medical University of Białystok, ul. Waszyngtona 13, 15-269 Białystok, Poland; 45026@student.umb.edu.pl (Z.R.); 43571@student.umb.edu.pl (K.S.); 45018@student.umb.edu.pl (J.N.); 45042@student.umb.edu.pl (M.C.); 45024@student.umb.edu.pl (M.P.); 45043@student.umb.edu.pl (K.D.); 45367@student.umb.edu.pl (P.M.)

**Keywords:** oral leukoplakia, saliva, oxidative stress, antioxidants, malondialdehyde, glutathione, 8-OHdG, uric acid, vitamins C and E, systematic review, meta-analysis

## Abstract

Oral leukoplakia (OL) is a common oral potentially malignant disorder in which chronic inflammation and carcinogenic exposures may promote oxidative stress. Saliva is in direct contact with the lesion and represents a non-invasive medium for assessing redox dysregulation. This systematic review and meta-analysis synthesized evidence on salivary oxidative damage markers and antioxidant defenses in OL compared with healthy controls. A PROSPERO-registered systematic review (CRD420251242238) was conducted in accordance with PRISMA and Cochrane guidelines. PubMed, Scopus and Web of Science were searched up to 10 December 2025 for observational studies comparing salivary oxidative stress and/or antioxidant markers in patients with clinically and/or histopathologically confirmed OL and healthy controls. Case–control and cross-sectional studies reporting quantitative data were included. Risk of bias was assessed using a modified Newcastle–Ottawa Scale. When ≥2 datasets were available, standardized mean differences (SMDs) with 95% confidence intervals (CI) were pooled. Meta-analysis showed significantly higher salivary malondialdehyde in OL (SMD = 1.47; 95% CI: 0.55–2.39), indicating enhanced lipid peroxidation. OL was also associated with significantly lower levels of reduced glutathione, vitamins C and E, and uric acid. For 8-hydroxy-2′-deoxyguanosine, a non-significant trend towards higher levels was observed with substantial heterogeneity. Evidence for TBARS, total antioxidant capacity and enzymatic antioxidants was limited. OL is associated with a salivary redox imbalance favoring a pro-oxidant state. High heterogeneity and limited biomarker-specific evidence highlight the need for larger, standardized prospective studies to validate salivary redox markers for OL monitoring and risk stratification.

## 1. Introduction

Oral leukoplakia (OL) is among the most common potentially malignant disorders of the oral mucosa and remains a major clinical concern because of its risk of malignant transformation [1,2,3]. According to World Health Organization (WHO) guidelines, OL is defined as a white oral mucosal lesion that cannot be attributed to any other disease entity based on clinical and histopathological findings and that is associated with an increased probability of cancer development [1,3]. This is therefore a diagnosis of exclusion, requiring differentiation from other white lesions such as oral lichen planus, candidiasis, traumatic lesions, leukoderma and chemical burns [2,3].

OL is the most prevalent condition within the group of oral potentially malignant disorders (OPMDs), alongside erythroplakia, proliferative verrucous leukoplakia and oral submucous fibrosis [4,5]. A meta-analysis including more than one million individuals reported a global prevalence of approximately 2.23% in population-based studies, while in high-risk groups (e.g., regular tobacco or alcohol users), prevalence may reach ~9%, underscoring its epidemiological relevance and the need for particular clinical attention [5,6].

The pathogenesis of OL remains complex: a key role is attributed to chronic exposure to irritants, leading to dysregulated epithelial proliferation, an abnormal inflammatory response, and the accumulation of mutations that promote the development of dysplasia [1,3]. The presence and severity of oral epithelial dysplasia (OED)—defined by architectural and cytological abnormalities—represent the principal prognostic factor [7]. WHO criteria classify OED as mild, moderate, or severe, with increasing grades associated with a higher risk of malignant transformation and the need for more intensive surveillance [5,8]. Recent publications also distinguish differentiated dysplasia, which may follow a different clinical course and prognosis [9]. The mean malignant transformation rate of OL is approximately 6.64%, with higher risk reported for non-homogeneous lesions, larger lesions, lesions with dysplasia, and in smokers [10].

Clinical management is guided by the grade of dysplasia—lesions without dysplasia or with mild dysplasia are typically monitored after elimination of risk factors (tobacco, alcohol, and chronic mechanical irritation), whereas moderate and severe dysplasia more often warrant definitive treatment, most commonly surgical excision [1,5,11]. Therapeutic approaches include scalpel excision, laser therapy, or ablation. Regardless of the method, long-term follow-up is required because the risk of recurrence and progression persists even after treatment [11,12].

One of the most frequently discussed processes that may promote malignant transformation in OPMDs, including OL, is oxidative stress [13,14]. It is defined as a state in which the production of reactive oxygen species (ROS) exceeds antioxidant defense capacity, resulting in oxidative damage to lipids, proteins, and nucleic acids [13,15,16,17,18,19,20]. In the oral cavity, ROS may be generated by free radicals in tobacco smoke, alcohol metabolism, chronic irritation, microbial activity, and inflammatory cells [21,22]. Although ROS serve signaling functions at low levels, their excess promotes lipid peroxidation, protein carbonylation, and oxidative DNA base modifications, thereby increasing mutational risk, supporting the clonal expansion of damaged cells, and activating pro-proliferative and pro-angiogenic signaling pathways [14,23,24]. Protection against ROS overload is provided by a complex antioxidant system that includes enzymatic mechanisms (e.g., superoxide dismutase (SOD), catalase (CAT), and glutathione peroxidase (GPx)) and non-enzymatic antioxidants (reduced glutathione (GSH), uric acid (UA), and vitamins C and E).

In assessing redox balance in oral diseases, saliva is increasingly used as a readily accessible diagnostic medium. It is produced by hundreds of minor submucosal glands as well as the major paired salivary glands: parotid, submandibular, and sublingual [25,26,27]. As a fluid in direct contact with mucosal lesions, saliva may reflect both local and systemic oxidative processes [16,18,20,26,28,29,30]. A wide range of salivary biomarkers is employed to characterize oxidative stress and antioxidant capacity, including lipid peroxidation products (malondialdehyde (MDA) and thiobarbituric acid reactive substances (TBARS)), markers of oxidative DNA damage (8-OHdG), antioxidant enzymes (SOD, CAT, GPx, and glutathione S-transferase), low-molecular-weight antioxidants (GSH, UA, and vitamins C and E), and composite indices (total oxidant status (TOS), total antioxidant capacity (TAC), and oxidative stress index (OSI)) [18,19,28,29,31,32,33,34]. The minimally invasive nature, ease of collection, and high patient acceptability make salivary redox markers attractive candidates for monitoring OPMDs in clinical practice [28,35].

Over the past two decades, several case–control studies have compared salivary oxidative stress markers and antioxidants in patients with OL and healthy controls. Most reports indicate increased levels of oxidative damage markers (e.g., MDA, 8-OHdG, TBARS) along with concomitant reductions in selected components of antioxidant defense (e.g., GSH, UA, vitamins C and E) [20,34,36,37,38,39,40,41,42,43]. However, studies differ substantially in sample size, demographic characteristics, severity of dysplastic changes, saliva collection procedures, and analytical methods, limiting comparability. Consequently, the direction and magnitude of the observed differences are not fully consistent across markers, and the overall salivary redox profile in OL remains unclear. Importantly, previous reviews have typically addressed heterogeneous OPMD groups, combined findings from different body fluids, or did not include formal quantitative synthesis via meta-analysis.

To our knowledge, no systematic review and meta-analysis has specifically and comprehensively quantified salivary oxidative stress and antioxidant markers in patients with oral leukoplakia compared with healthy individuals. Therefore, the aim of this study was to quantitatively synthesize evidence from observational studies on differences in salivary oxidative stress markers and antioxidant capacity between patients with OL and healthy controls and, where data permitted, to evaluate their associations with selected clinico-pathological features of OL, including the grade of epithelial dysplasia and lesion size.

## 2. Materials and Methods

### 2.1. Search Strategy, Eligibility Criteria and Data Extraction

The protocol was registered in PROSPERO (CRD420251242238), and the review was conducted in accordance with the Cochrane Handbook and PRISMA guidelines [44,45]. The review focused on alterations in salivary oxidative stress and antioxidant markers in patients with oral leukoplakia compared with systemically healthy controls.

To identify all relevant evidence on salivary redox imbalance in oral leukoplakia, we conducted a systematic literature search in three electronic databases: PubMed, Scopus and Web of Science. Google Scholar was not used as a primary source because of its limited transparency and inconsistent indexing. Detailed search strategies for each database are presented below.

1. For PubMed: (leukoplak* OR leucoplak* OR oral leukoplakia OR OLK OR OL) AND (saliv* OR oral fluid* OR whole saliva OR oral secretion* OR mouthrinse OR oral rinse OR oral wash OR oral swab OR oral mucosal transudate) AND (oxidat* OR antioxid* OR redox OR oxidative stress OR oxidation-reduction OR oxidant* OR prooxidant* OR reactive oxygen species OR ROS OR reactive nitrogen species OR RNS OR nitrosat* OR superoxide OR hydrogen peroxide OR peroxide* OR lipid peroxidation OR isoprostane* OR 8-iso-prostaglandin F2alpha OR malondialdehyde OR MDA OR TBARS OR 4-hydroxynonenal OR 4-HNE OR protein carbonyl* OR advanced oxidation protein products OR AOPP OR total antioxidant capacity OR TAC OR FRAP OR TEAC OR CUPRAC OR ABTS OR DPPH OR ORAC OR TRAP OR total oxidant status OR TOS OR oxidative stress index OR OSI OR d-ROMs OR derivatives of reactive oxygen metabolites OR 8-OHdG OR 8-hydroxy-2′-deoxyguanosine OR 8-oxodG OR 8-oxo-2′-deoxyguanosine OR nitrotyrosine OR NOx OR nitric oxide OR SOD OR superoxide dismutase OR catalase OR CAT OR GPx OR glutathione peroxidase OR peroxidase OR MPO OR myeloperoxidase OR PON1 OR paraoxonase OR GR OR glutathione reductase OR GST OR glutathione S-transferase OR glutathione OR GSH OR GSSG OR uric acid OR ascorbate OR vitamin C OR tocopherol OR vitamin E OR carotenoid* OR biomarker* OR marker*)
2. For Scopus: TITLE-ABS-KEY ((leukoplak* OR leucoplak* OR “oral leukoplakia” OR OLK OR OL) AND (saliv* OR “oral fluid*” OR “whole saliva” OR “oral secretion*” OR mouthrinse OR “oral rinse” OR “oral wash” OR “oral swab” OR “oral mucosal transudate”) AND (oxidat* OR antioxid* OR redox OR “oxidative stress” OR “oxidation-reduction” OR oxidant* OR prooxidant* OR “reactive oxygen species” OR ROS OR “reactive nitrogen species” OR RNS OR nitrosat* OR superoxide OR “hydrogen peroxide” OR peroxide* OR “lipid peroxidation” OR isoprostane* OR “8-iso-prostaglandin F2alpha” OR malondialdehyde OR MDA OR TBARS OR “4-hydroxynonenal” OR 4-HNE OR “protein carbonyl*” OR “advanced oxidation protein products” OR AOPP OR “total antioxidant capacity” OR TAC OR FRAP OR TEAC OR CUPRAC OR ABTS OR DPPH OR ORAC OR TRAP OR “total oxidant status” OR TOS OR “oxidative stress index” OR OSI OR “d-ROMs” OR “derivatives of reactive oxygen metabolites” OR 8-OHdG OR “8-hydroxy-2′-deoxyguanosine” OR 8-oxodG OR “8-oxo-2′-deoxyguanosine” OR nitrotyrosine OR NOx OR “nitric oxide” OR SOD OR “superoxide dismutase” OR catalase OR CAT OR GPx OR “glutathione peroxidase” OR peroxidase OR MPO OR myeloperoxidase OR PON1 OR paraoxonase OR GR OR “glutathione reductase” OR GST OR “glutathione S-transferase” OR glutathione OR GSH OR GSSG OR “uric acid” OR ascorbate OR “vitamin C” OR tocopherol OR “vitamin E” OR carotenoid* OR biomarker* OR marker*))
3. For Web of Science: TS = ((leukoplak* OR leucoplak* OR “oral leukoplakia” OR OLK OR OL) AND (saliv* OR “oral fluid*” OR “whole saliva” OR “oral secretion*” OR mouthrinse OR “oral rinse” OR “oral wash” OR “oral swab” OR “oral mucosal transudate”) AND (oxidat* OR antioxid* OR redox OR “oxidative stress” OR “oxidation-reduction” OR oxidant* OR prooxidant* OR “reactive oxygen species” OR ROS OR “reactive nitrogen species” OR RNS OR nitrosat* OR superoxide OR “hydrogen peroxide” OR peroxide* OR “lipid peroxidation” OR isoprostane* OR “8-iso-prostaglandin F2alpha” OR malondialdehyde OR MDA OR TBARS OR “4-hydroxynonenal” OR 4-HNE OR “protein carbonyl*” OR “advanced oxidation protein products” OR AOPP OR “total antioxidant capacity” OR TAC OR FRAP OR TEAC OR CUPRAC OR ABTS OR DPPH OR ORAC OR TRAP OR “total oxidant status” OR TOS OR “oxidative stress index” OR OSI OR “d-ROMs” OR “derivatives of reactive oxygen metabolites” OR 8-OHdG OR “8-hydroxy-2′-deoxyguanosine” OR 8-oxodG OR “8-oxo-2′-deoxyguanosine” OR nitrotyrosine OR NOx OR “nitric oxide” OR SOD OR “superoxide dismutase” OR catalase OR CAT OR GPx OR “glutathione peroxidase” OR peroxidase OR MPO OR myeloperoxidase OR PON1 OR paraoxonase OR GR OR “glutathione reductase” OR GST OR “glutathione S-transferase” OR glutathione OR GSH OR GSSG OR “uric acid” OR ascorbate OR “vitamin C” OR tocopherol OR “vitamin E” OR carotenoid* OR biomarker* OR marker*))

The first search was conducted on 6 December 2025, and the last on 10 December 2025. We aimed to retrieve observational studies comparing salivary oxidative stress and antioxidant biomarkers between patients with clinically and/or histopathologically confirmed oral leukoplakia and systemically healthy controls. No restrictions on year of publication were applied, but the search was limited to articles published in English and involving human participants. We excluded studies conducted exclusively on serum or other biological fluids, non-comparative designs, case reports, reviews, conference abstracts, editorials, experimental animal or in vitro studies, and articles with insufficient quantitative data for extraction or meta-analysis.

Titles and abstracts were independently screened by two reviewers (Z.R. and K.S.), followed by full-text assessment to determine eligibility based on the predefined PECO-based inclusion and exclusion criteria for salivary redox markers in oral leukoplakia (Table 1). Any disagreements were resolved by discussion and, when consensus could not be reached, by consultation with a third reviewer (P.W.). Potential duplicate records were identified and removed using Zotero 7.0.30 reference management software by both reviewers (Z.R. and K.S.).

For studies that did not provide sufficient quantitative information, we attempted to contact the corresponding authors to obtain the original data. If no response was received or the necessary data could not be retrieved, the study was excluded from the quantitative synthesis.

From each eligible study, we extracted:

1. Article-level information—first author, year of publication, country, journal, study design, and sample size;
2. Participant characteristics—age and sex;
3. Outcome data—salivary oxidative stress and antioxidant biomarkers reported as mean ± SD or median with IQR for both leukoplakia patients and controls, sample sizes, and details of the saliva collection protocol and analytical methods.

### 2.2. Quality Assessment

The methodological quality and risk of bias of observational studies were independently assessed by two reviewers (Z.R. and K.S.) using a modified Newcastle–Ottawa Scale (NOS), which covers three domains—the selection of study groups, comparability of cases and controls, and ascertainment of exposure/outcome [46]. Each fulfilled item was awarded one star, yielding a maximum of nine stars per study. Discrepancies were resolved through discussion and, when necessary, consultation with a third reviewer (P.W.). In line with commonly used thresholds, studies scoring 7–9 stars were considered high quality (low risk of bias), those with 4–6 stars as moderate quality, and those with 0–3 stars as low quality (high risk of bias).

### 2.3. Statistical Analysis

Once at least two independent studies reported the same salivary biomarker in patients with oral leukoplakia and healthy controls, that marker was considered eligible for quantitative synthesis. For each comparison, effect sizes were calculated from the reported sample size and mean ± standard deviation (SD) in the leukoplakia and control groups. Because of expected clinical and methodological differences between studies, all effects were expressed as standardized mean differences (SMDs) with corresponding 95% confidence intervals (95% CI). A two-sided *p* value < 0.05 was regarded as statistically significant.

When continuous data were presented as median with interquartile range (IQR) or as median with minimum and maximum values, the corresponding mean and SD were approximated using published formulas by Wan et al. (2014) and Luo et al. (2018) [47,48]. Statistical heterogeneity across studies was quantified using the I^2^ statistic, with values of approximately 25%, 50%, and 75% interpreted as low, moderate, and high heterogeneity, respectively. A random-effects model was applied when heterogeneity was substantial (I^2^ > 50%); otherwise, a fixed-effect model was used. For meta-analyses including only two studies, a fixed-effect approach was prespecified, as between-study variance (τ^2^) cannot be reliably estimated with such sparse data.

Sensitivity analyses were performed using a leave-one-out approach, sequentially omitting each study and recalculating the pooled SMD. Because none of the biomarker-specific meta-analyses included ten or more studies, formal meta-regression analyses were not conducted, in line with methodological recommendations. However, for salivary MDA, exploratory analyses were performed to examine whether the analytical method used for MDA determination (TBARS-based spectrophotometric assays versus ELISA-based methods) influenced pooled effect estimates. These analyses were considered hypothesis-generating and were interpreted cautiously.

Because of the limited number of studies per outcome, publication bias was not formally assessed using funnel plots [49]. All statistical analyses, including meta-analyses, sensitivity analyses, and exploratory method-based comparisons, were performed using the MAJOR meta-analysis module in jamovi (version 2.7.13).

The overall certainty of evidence was assessed using the Grading of Recommendations Assessment, Development and Evaluation (GRADE) approach, applied to the body of evidence as a whole rather than to individual biomarkers [50].

## 3. Results

### 3.1. Literature Searches

**Figure 1:**
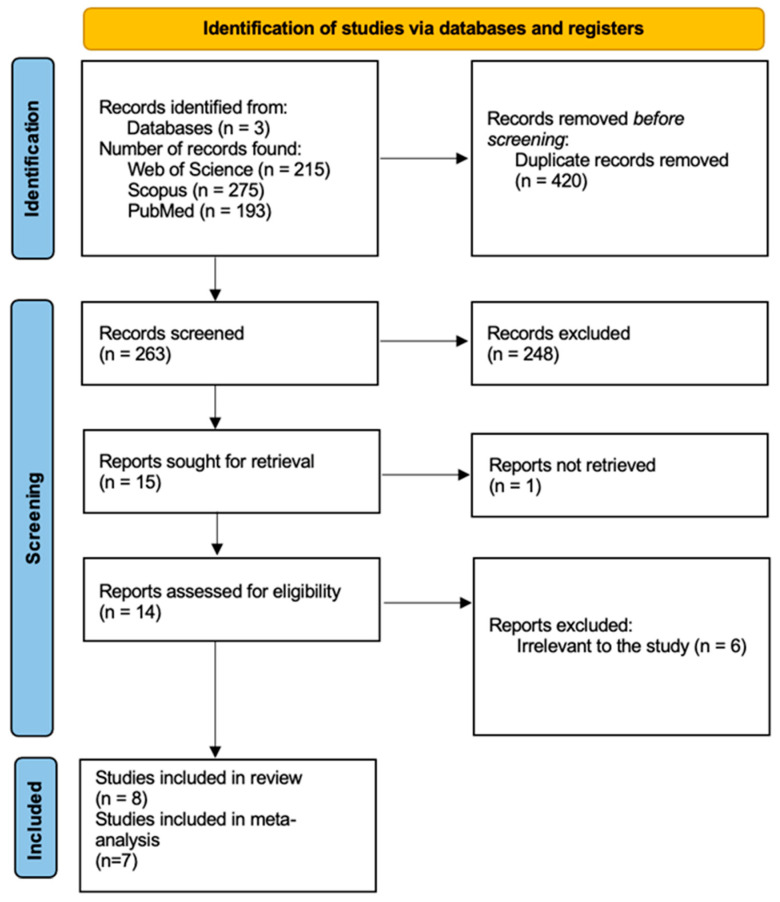
PRISMA flow chart (accessed on 10 December 2025).

The study selection process is summarized in the PRISMA flow diagram (Figure 1). The electronic search of PubMed, Scopus and Web of Science identified a total of 683 records up to 10 December 2025 (193 from PubMed, 275 from Scopus and 215 from Web of Science). After the removal of 420 duplicate records, 263 unique records remained for title and abstract screening. Of these, 248 were excluded as clearly irrelevant (e.g., studies not involving oral leukoplakia, absence of salivary sampling, no assessment of oxidative stress/antioxidant biomarkers, animal or in vitro studies, reviews, or non-English publications).

Fifteen full-text articles were sought for retrieval. One report could not be obtained despite attempts to access the full text. Fourteen full-text articles were assessed for eligibility, and six were excluded because they did not meet the predefined PECO criteria. Ultimately, eight studies fulfilled the inclusion criteria and were included in the qualitative synthesis. However, because one study did not report sufficiently detailed quantitative data, only seven studies could be included in the meta-analysis.

### 3.2. Study Characteristics

A total of eight eligible studies are summarized in detail in Table 2. Altogether, these case–control studies included 209 patients with oral leukoplakia and 211 systemically healthy controls. For the quantitative synthesis, we considered six salivary redox biomarkers that were reported in at least two independent datasets: the lipid peroxidation product MDA, the DNA oxidation marker 8-OHdG, and four antioxidant parameters (GSH, uric acid, vitamin C and vitamin E). Table 3 summarizes available data on key clinical and exposure-related characteristics, including smoking status, presence and grade of epithelial dysplasia, and lesion localization. Methodological aspects of saliva sampling and analysis—such as saliva type, collection protocols and pre-analytical conditions—are presented in Table 4.

Table 5 provides an overview of the meta-analytic results for each biomarker, including the number of contributing studies and participants, the pooled standardized mean difference with 95% confidence intervals, and the corresponding heterogeneity statistics (Q, *p* value and I^2^).

**Figure 2:**
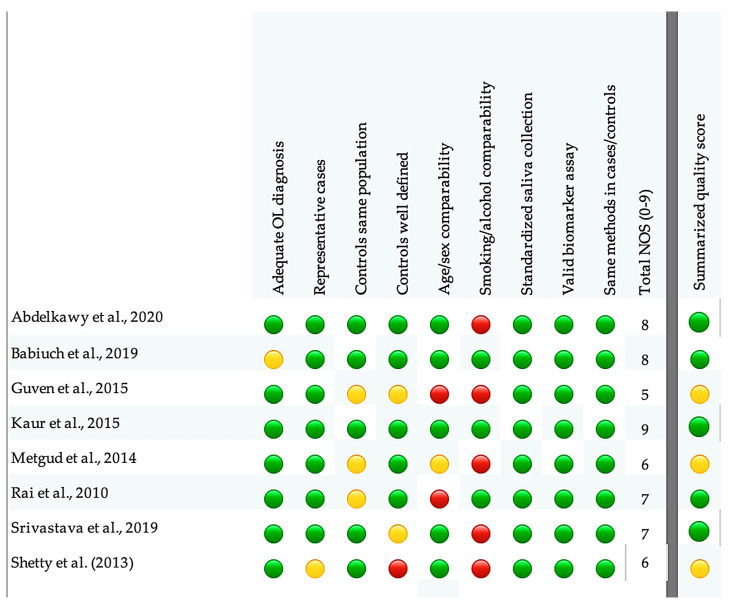
Risk of bias assessment [34,36,38,39,40,41,42,43].

All included studies used a case–control design, and patients were diagnosed with oral leukoplakia on clinical and, in most reports, histopathological grounds. Newcastle–Ottawa Scale scores ranged from 5 to 9 (Table 2). According to our predefined thresholds, three studies (5–6 points) were judged to have moderate methodological quality, whereas the remaining five studies (7–9 points) were considered high quality; none of the studies was classified as low quality (Figure 2).

### 3.3. Oxidative Stress Markers in OL

#### 3.3.1. Malondialdehyde (MDA)

Based on the six case–control studies that assessed salivary MDA in oral leukoplakia, a total of 144 OL patients and 146 healthy controls were included in the quantitative synthesis (Table 3) [34,36,38,40,41,42].

**Figure 3:**
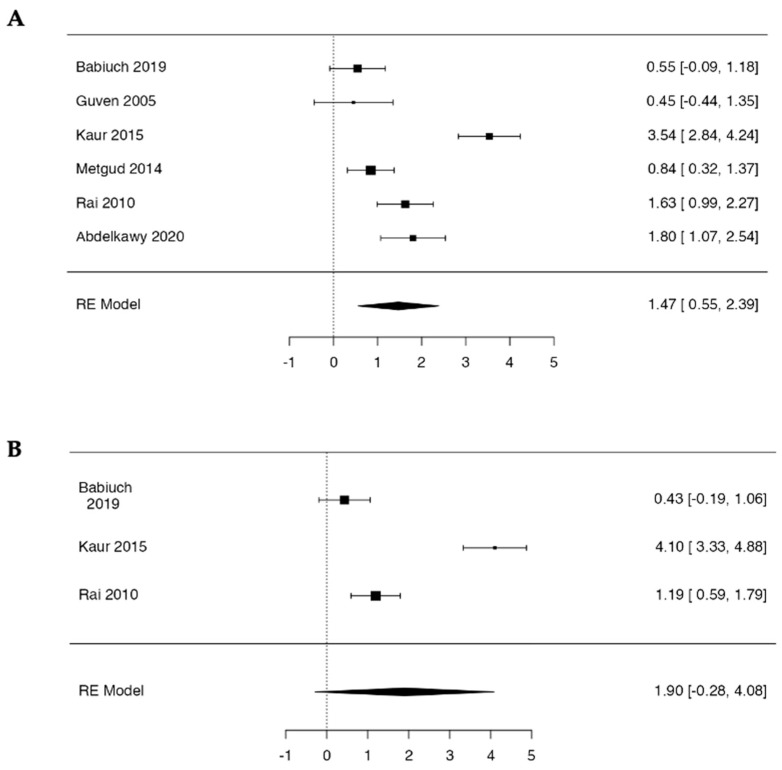
Forest plots of the random-effects meta-analysis of salivary oxidative stress markers: (**A**) salivary MDA [34,36,38,40,41,42] and (**B**) salivary 8-OHdG [36,38,42].

In all datasets, mean MDA concentrations were higher in the leukoplakia group than in controls. The random-effects meta-analysis showed a large pooled standardized mean difference of 1.47 (95% CI 0.55 to 2.39; Z = 3.13, *p* = 0.002), indicating substantially increased salivary MDA levels in OL. Between-study variability was considerable (Q = 52.86, df = 5, *p* < 0.001; I^2^ = 91%), consistent with pronounced heterogeneity in effect sizes across individual studies (Figure 3).

#### 3.3.2. 8-Hydroxy-2′-deoxyguanosine (8-OHdG)

Salivary 8-hydroxy-2′-deoxyguanosine (8-OHdG) was evaluated in three case–control studies, comprising 85 patients with oral leukoplakia and an equal number of healthy controls (Table 3) [36,38,42]. All studies reported higher mean 8-OHdG concentrations in the leukoplakia group, although not all individual comparisons reached statistical significance.

In the random-effects meta-analysis, the pooled standardized mean difference was 1.90 (95% CI −0.28 to 4.08; Z = 1.71, *p* = 0.088), indicating a non-significant overall trend toward increased salivary 8-OHdG in oral leukoplakia. Between-study heterogeneity was extreme (Q = 32.5, df = 2, *p* < 0.001; I^2^ = 96.9%), reflecting large variability in effect sizes across the included datasets (Figure 3).

#### 3.3.3. Others

Srivastava et al. (2019) also assessed salivary thiobarbituric acid-reactive substances (TBARS) as a global marker of lipid peroxidation in oral leukoplakia [39]. In this study (40 OL vs. 40 healthy controls), TBARS levels were substantially higher in the leukoplakia group, with a highly significant difference between groups (*p* < 0.001). As TBARS was reported in only one study and reflects a composite TBA-reactive signal, these data were summarized qualitatively and were not included in the meta-analysis.

### 3.4. Antioxidants Activity

#### 3.4.1. Reduced Glutathione (GSH)

Reduced glutathione concentration in saliva was analyzed in two case–control studies, including 50 patients with oral leukoplakia and 50 healthy controls (Table 3) [34,36]. In both datasets, mean GSH levels were lower in the leukoplakia group than in controls.

**Figure 4:**
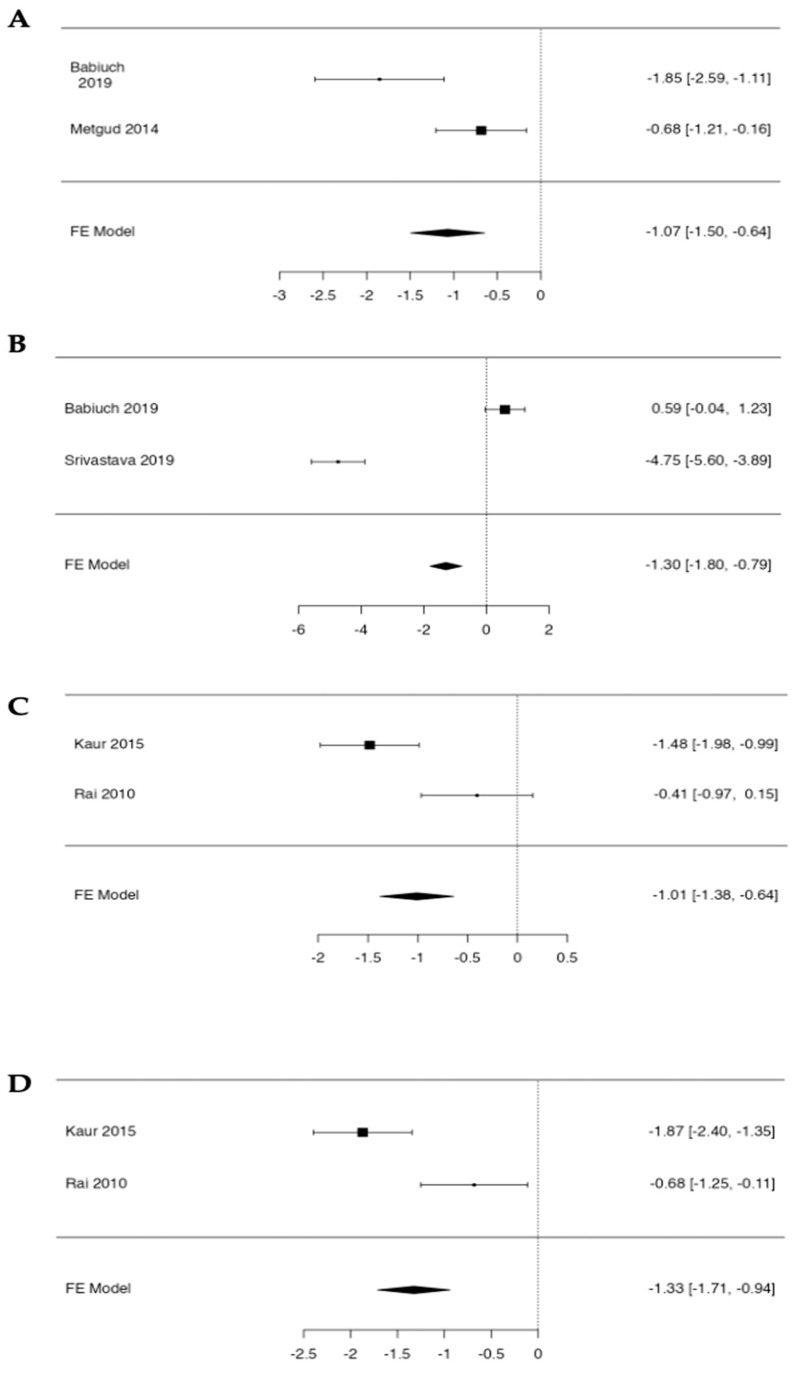
Forest plots of the fixed-effects meta-analysis of salivary antioxidant markers: (**A**) GSH [34,36], (**B**) uric acid (UA) [36,39], (**C**) vitamin C [38,42], and (**D**) vitamin E [38,42].

The fixed-effects meta-analysis confirmed a significant decrease in salivary GSH in OL, with a pooled standardized mean difference of −1.07 (95% CI −1.50 to −0.65; Z = −4.93, *p* < 0.001). Heterogeneity between the two studies was considerable (Q = 6.39, df = 1, *p* = 0.011; I^2^ = 84.4%), indicating notable differences in the magnitude of GSH depletion across studies (Figure 4).

#### 3.4.2. Uric Acid (UA)

Salivary uric acid was reported in two case–control studies, including 60 patients with oral leukoplakia and 60 healthy controls (Table 3) [36,39]. Both datasets showed lower mean UA concentrations in the leukoplakia group than in controls.

The fixed-effects meta-analysis confirmed a significant reduction in salivary uric acid in OL, with a pooled standardized mean difference of −1.30 (95% CI −1.81 to −0.79; Z = −4.99, *p* < 0.001). However, the between-study heterogeneity was extremely high (Q = 96.55, df = 1, *p* < 0.001; I^2^ = 98.96%), indicating substantial inconsistency in the magnitude of UA depletion across the two available studies (Figure 4).

#### 3.4.3. Vitamin C

Salivary vitamin C was evaluated in two case–control studies, comprising 65 oral leukoplakia patients and 65 healthy controls (Table 3) [38,42]. In both datasets, mean vitamin C levels were lower in the OL group.

The pooled analysis showed a significant decrease in salivary vitamin C, with a standardized mean difference of −1.01 (95% CI −1.38 to −0.65; Z = −5.34, *p* < 0.001). Heterogeneity between the two studies was high (Q = 7.98, df = 1, *p* = 0.005; I^2^ = 87.5%), suggesting notable variability in the extent of vitamin C depletion across studies (Figure 4).

#### 3.4.4. Vitamin E

Salivary vitamin E was also investigated in two case–control studies, including 65 patients with oral leukoplakia and 65 healthy controls (Table 3) [38,42]. Both studies reported lower vitamin E concentrations in the OL group.

The meta-analysis demonstrated a marked reduction in salivary vitamin E, with a pooled standardized mean difference of −1.33 (95% CI −1.71 to −0.94; Z = −6.72, *p* < 0.001). As with vitamin C, heterogeneity was high (Q = 9.03, df = 1, *p* = 0.003; I^2^ = 88.9%), indicating considerable differences in the size of the effect between the two studies (Figure 4).

#### 3.4.5. Others

Only one case–control study evaluated salivary glutathione S-transferase (GST) in oral leukoplakia. In the study by Srivastava et al. (2019), 40 leukoplakia patients were compared with 40 healthy controls, and the mean GST activity was substantially lower in the leukoplakia group (*p* < 0.001) [39]. As GST was reported in a single dataset, this marker was summarized qualitatively and was not included in the meta-analysis.

Salivary superoxide dismutase (SOD) was assessed in two studies. In the three-arm study by Shetty et al. (2013), SOD activity was highest in healthy controls, intermediate in leukoplakia and lowest in oral squamous cell carcinoma, with a significant reduction in leukoplakia compared with controls (*p* = 0.01) [43]. In contrast, in the multi-marker case–control study by Babiuch et al. (2019), SOD levels did not differ significantly between leukoplakia and healthy subjects, while being increased only in the oral cancer group [36]. Because dispersion measures required to derive effect sizes were not fully reported in Shetty et al. (2013), and the two studies yielded inconsistent findings, SOD was not synthesized quantitatively and its results are presented descriptively [43].

In the same study by Babiuch et al. (2019), several additional antioxidant indices were examined, including total antioxidant capacity (TAC), glutathione peroxidase (GPx), glutathione reductase (GR), total glutathione (tGSH), oxidized glutathione (GSSG) and the GSH/GSSG ratio [36]. Among the markers that were not entered into our meta-analyses, only the GSH/GSSG ratio was significantly lower in oral leukoplakia than in controls, whereas TAC, GPx, GR, tGSH and GSSG showed no significant group differences. As these parameters were available from a single study only, they were not pooled quantitatively and are reported as exploratory findings.

### 3.5. Salivary Redox Markers in Relations to OL Severity

Two studies examined how salivary redox markers vary with the histopathological grade of oral leukoplakia. In the study by Srivastava et al. (2019), TBARS showed a significant stepwise increase from mild through moderate to severe dysplasia, whereas the antioxidant markers GST and uric acid decreased progressively with advancing grades [39]. All trends were statistically significant, suggesting that more severe dysplasia is associated with higher lipid peroxidation and a weaker salivary antioxidant defense. In contrast, Metgud et al. (2014) did not detect significant differences in salivary MDA or GSH concentrations across mild, moderate and severe dysplasia within the leukoplakia group—mean values for both markers were similar between grades [34].

One study explored whether salivary redox markers vary with the clinical size of oral leukoplakia. Babiuch et al. (2019), patients with larger leukoplakia lesions (2–4 cm in greatest dimension) showed significantly higher total antioxidant capacity (TAC) than those with smaller lesions (<2 cm) [36]. In contrast, no clear size-dependent differences were observed for the other measured biomarkers, including SOD, GSH, tGSH, GSSG, GPx, GR, uric acid, 8-OHdG and MDA. Moreover, in the same cohort, the epithelial dysplasia grade was not associated with the level of any salivary biomarker.

### 3.6. Subgroup Analysis

Subgroup analyses were conducted for salivary MDA studies according to the analytical method used for biomarker determination (TBARS-based spectrophotometric assays vs. ELISA-based methods). The pooled effect estimates for each subgroup are presented in Table 4. Both assay-based subgroups demonstrated higher salivary MDA levels in patients with oral leukoplakia compared with healthy controls. However, no statistically significant difference in effect size was observed between TBARS-based and ELISA-based studies (*p* for interaction = 0.676). Substantial heterogeneity persisted within both subgroups, indicating that assay methodology alone did not explain the observed between-study variability (Table 6).

### 3.7. Sensitivity Analysis

In a leave-one-out sensitivity analysis for salivary MDA, sequential exclusion of each individual study did not materially change the magnitude or significance of the pooled effect, which remained in the SMD range of 1.06–1.66. Heterogeneity also persisted at a high level (I^2^ ≈ 66–92%), indicating that no single study was solely responsible for the observed between-study variability (Table 7).

### 3.8. Certainty of Evidence (GRADE Assessment)

The GRADE assessment indicated very low certainty of evidence for the association between oral leukoplakia and alterations in salivary redox biomarkers. Although several pooled effects were large and directionally consistent, the certainty was downgraded for risk of bias, inconsistency, and imprecision. Risk of bias was considered serious because the evidence was derived exclusively from observational case–control studies and because control of key confounders (particularly smoking and other factors influencing salivary redox status) was inconsistent across studies, despite overall moderate-to-high NOS ratings (5–9 points). Inconsistency was judged as serious due to substantial to extreme heterogeneity in most meta-analyses, including MDA (I^2^ = 91.1%), 8-OHdG (I^2^ = 96.9%), and the antioxidant markers synthesized from two studies each (e.g., GSH I^2^ = 84.4%, uric acid I^2^ = 99.0%, vitamin C I^2^ = 87.5%, vitamin E I^2^ = 88.9%), indicating marked variability in effect magnitude across datasets. Imprecision was also rated serious because several outcomes were informed by only two or three studies with limited total sample sizes, and—for some markers—confidence intervals remained wide or crossed the line of no effect (e.g., 8-OHdG: SMD = 1.90, 95% CI −0.28 to 4.08). In addition, no clinically established decision thresholds for salivary redox biomarkers in oral leukoplakia are available, which further limits confidence in the precision and applicability of pooled estimates for practice. Publication bias could not be formally assessed because each meta-analysis included fewer than ten studies; therefore, it remains uncertain. Overall, while the pooled estimate for MDA suggested a marked increase in salivary lipid peroxidation in oral leukoplakia (SMD = 1.47, 95% CI 0.55 to 2.39), the GRADE rating remained very low due to the observational design, substantial heterogeneity, and limited information size. Domain-level GRADE judgments are provided in Supplementary File S2.

### 3.9. Summary of the Findings

To provide a concise overview of the results, the direction of changes in all assessed salivary oxidative stress and antioxidant biomarkers in patients with oral leukoplakia compared with healthy controls is summarized in Table 8. The table distinguishes between biomarkers supported by meta-analytic evidence and those reported only qualitatively, allowing rapid reference to the overall pattern of findings.

## 4. Discussion

This systematic review and meta-analysis indicate that OL is associated with a marked shift in salivary redox balance toward a pro-oxidant state. This pattern comprises a concomitant increase in biomarkers of oxidative damage—particularly lipid peroxidation—and impairment of multi-layered antioxidant defense mechanisms, including both enzymatic and non-enzymatic components.

Oxidative stress is defined as a state in which reactive oxygen species production exceeds the compensatory capacity of antioxidant systems, resulting in damage to DNA, lipids, and proteins and the disruption of redox homeostasis [51,52,53,54,55]. In oral leukoplakia, chronic oxidative stress is closely linked to persistent inflammation and is considered a relevant factor in premalignant progression [13,53,56,57,58,59,60,61]. Prolonged exposure of epithelial cells to excessive ROS promotes DNA damage, genomic instability, and the selection of clones with proliferative advantage, thereby increasing the risk of malignant transformation [57,62,63,64,65,66]. From this perspective, the assessment of salivary oxidative stress biomarkers reflects biological processes involved in the progression of premalignant lesions rather than merely overall oxidative burden. Elevated lipid peroxidation and oxidative DNA damage markers, together with reduced endogenous antioxidant levels, indicate a persistent disruption of redox homeostasis within the mucosal microenvironment [34,35,39,56,67].

The most consistent quantitative finding of our meta-analysis was a significant increase in salivary malondialdehyde (MDA) levels in patients with OL compared with healthy controls, supported by the pooled effect estimate (SMD = 1.47; 95% CI: 0.55–2.39) and accompanied by substantial heterogeneity (I^2^ = 91.1%). Lipid peroxidation may proceed through enzymatic pathways catalyzed, among others, by lipoxygenases (LOX) and cyclooxygenases (COX), as well as through non-enzymatic reactions initiated by free radicals [68,69]. In this chain reaction, ROS attack lipid double bonds—particularly those of polyunsaturated fatty acids—leading to the formation of lipid radicals and lipid hydroperoxides [68,70,71]. The cumulative intensity of these processes is most commonly assessed using MDA concentrations and thiobarbituric acid reactive substance (TBARS) values [34,72,73,74,75,76,77]. MDA is a key product of lipid peroxidation with high biological reactivity; it can react with DNA and proteins to form adducts that disrupt enzyme function, modulate gene expression, and induce mutations [68,69,70,71]. Interactions of MDA with macromolecules may disrupt genomic stability and gene regulation related to cell cycle control, proliferation, and apoptosis, leading to promutagenic modifications such as M1G [78,79,80,81,82,83,84,85]. Accordingly, TBARS may reflect not only the extent of lipid peroxidation but also mutagenic risk in oral leukoplakia [33,86,87]. The consistent direction of MDA changes across studies and the robustness of findings in sensitivity analyses support enhanced lipid peroxidation as a characteristic feature of oral leukoplakia, although substantial heterogeneity warrants caution when interpreting effect magnitude [36,38,40,41].

The diagnostic value of MDA assessment is complemented by more “global” lipid peroxidation indices such as TBARS. The TBARS assay, based on the reaction of thiobarbituric acid with MDA and other lipid-derived aldehydes, provides a non-specific but sensitive measurement of the cumulative pool of lipid peroxidation products [33,88]. Because TBARS captures not only MDA but also other reactive aldehydes, it may better reflect global oxidative status than isolated MDA measurement [33,39,89]. In the study by Srivastava et al. (2019), salivary TBARS levels were higher than in controls and increased with the severity of epithelial dysplasia, suggesting that lipid membrane peroxidation may intensify during premalignant progression, potentially increasing epithelial exposure to promutagenic aldehydes that—according to the literature—may promote DNA damage and mutagenesis [39]. However, the limited number of OL studies using TBARS precludes conclusions regarding the strength and reproducibility of this association comparable to those for MDA.

Oxidative stress in OL also involves damage to genetic material. One of the best-characterized biomarkers of oxidative DNA damage is 8-hydroxy-2′-deoxyguanosine (8-OHdG), whose biological relevance in carcinogenesis has been confirmed in in vitro and in vivo studies. 8-oxoG is a common and biologically relevant product of guanine oxidation formed under conditions of increased oxidative stress, including exposure to tobacco smoke and epithelial metabolic activity [90,91,92,93,94,95,96,97,98]. Because guanine is highly susceptible to oxidative modification, 8-oxoG is considered a mutagenic lesion capable of inducing G:C→T:A transversion mutations, thereby contributing to the dysregulation of genes involved in proliferation and differentiation [99,100,101,102,103]. Under physiological conditions, 8-oxoG is removed via base excision repair by OGG1, generating free 8-OHdG that can be released into body fluids. Salivary 8-OHdG levels have been shown to correlate with local oxidative tissue damage in the oral cavity [94,95,102,104,105].

In our meta-analysis, a trend toward higher salivary 8-OHdG levels was observed in OL patients (SMD = 1.9; 95% CI: −0.28–4.08), although the effect did not reach statistical significance and heterogeneity was very high [36,38,42]. Despite quantitative limitations and the exploratory nature of this synthesis, the direction of the observation is consistent with a mechanism in which chronic oxidative stress involves not only lipids but also epithelial DNA, increasing the burden on repair systems and potentially facilitating the persistence of molecular alterations.

Alongside increased damage markers, impaired and fragmented defense systems were evident, encompassing both the glutathione axis and other non-enzymatic antioxidant components of saliva [13,15,21,22,34,36,37,38,39]. The meta-analysis demonstrated a significant reduction in glutathione levels (SMD = −1.07; 95% CI: −1.497 to −0.645) [34,36]. GSH is a central thiol buffer and one of the most important regulators of redox homeostasis in stratified squamous epithelial cells: it neutralizes lipid hydroperoxides and hydrogen peroxide via the glutathione peroxidase (GPx) system, binds reactive aldehydes and xenobiotics via glutathione transferases, and helps maintain numerous proteins in their reduced form [106,107,108,109]. A decrease in the GSH/GSSG ratio indicates a shift toward oxidation and reduced buffering capacity against oxidative stress; oxidation of -SH groups may result in loss of protein activity, disturbed proliferation, amplified inflammation, and DNA damage promoting mutation accumulation [110,111,112,113]. Babiuch et al. (2019) reported a significantly lower GSH/GSSG ratio in OL patients, suggesting that GSH regeneration mechanisms may be insufficient within the OL microenvironment [36]. Persistent reduction in redox potential favors activation of oxidative stress response pathways such as Nrf2-Keap1 and NF-κB, which can induce protective gene expression; however, chronic activation may promote survival, proliferation, and adaptation of dysplastic cells to pro-apoptotic stimuli, consistent with the carcinogenesis “paradox” in which moderate but persistent oxidative stress selects for cells more resistant to damage [114,115,116,117].

Several studies have attempted to link redox disturbances with clinico-pathological features of OL. Srivastava et al. (2019) demonstrated increasing TBARS and decreasing GST and UA with greater dysplasia severity, consistent with a model in which advanced dysplasia is associated with higher mitotic activity, a stronger inflammatory infiltrate, and increased ROS production, leading to cumulative oxidative damage to lipids, proteins, and DNA [22,39,51]. In contrast, Metgud et al. (2014) found no significant differences in MDA or GSH between dysplasia grades, and Babiuch et al. (2019) reported no clear association between salivary biomarkers and histopathological grading [34,36]. These discrepancies may reflect small subgroup sizes, differences in classification criteria, and heterogeneity of clinical OL variants, as well as insufficient control of confounders such as smoking, alcohol use, periodontal disease, and other chronic oral inflammatory conditions, which can independently modify the salivary redox profile [17,22,39]. It is also possible that the salivary oxidative–antioxidative profile reflects the overall oxidative burden in the oral cavity and the extent/biological activity of lesions, rather than local dysplasia severity assessed from a single biopsy specimen, which could explain the lack of consistent correlations across studies.

Superoxide dismutase (SOD) constitutes a key first-line enzymatic defense against superoxide radicals by catalyzing their conversion to hydrogen peroxide and oxygen, thereby limiting secondary ROS formation [118,119,120,121,122]. Its activity is particularly relevant in mitochondria, where excess superoxide can enhance oxidative damage and redox-dependent inflammatory signaling. Chronic oxidative stress may result in functional overload and oxidative inactivation of SOD, promoting superoxide accumulation, lipid peroxidation, and the activation of pro-inflammatory signaling pathways [120,123,124,125,126,127,128,129,130,131,132,133,134,135,136,137].

Despite the strong biological rationale, evidence on salivary SOD activity in OL could not be quantitatively synthesized. Two studies assessed SOD—however, Shetty et al. (2013) did not report sufficient numerical data for statistical calculations (missing standard deviations), and thus results for this biomarker could only be summarized qualitatively [43]. Shetty et al. (2013) observed a gradual decrease in SOD activity from healthy controls through OL to oral squamous cell carcinoma (OSCC), which may indicate an increasing overload of enzymatic defenses with disease progression [43]. Conversely, Babiuch et al. (2019) found no difference in SOD activity between OL and controls but reported increased SOD activity in OSCC; a similar direction of increased antioxidant enzyme activity in oral cancer has also been described in other studies [36]. These discrepancies may be interpreted in terms of dynamic redox regulation: in OL, overload and/or inactivation of enzymatic defenses may predominate, whereas in OSCC, a secondary adaptation—partly Nrf2-Keap1-dependent—may allow ROS to be maintained at levels supporting redox signaling and the survival of transformed cells [138,139,140,141,142,143]. Moreover, OL heterogeneity and limitations inherent to salivary SOD measurement—which may not reflect intracellular enzyme activity—may further contribute to inconsistent observations [26,36,43,144]. Practically, this implies that although SOD has strong pathophysiological plausibility, the current evidence base in OL remains inconclusive and requires larger studies with standardized methodology and complete reporting enabling quantitative synthesis [36,43,118,119,120].

An important component of antioxidant defense is also provided by low-molecular-weight, non-enzymatic salivary antioxidants, which act as an initial “redox buffer” neutralizing ROS before they interact with epithelial cells [145,146]. The meta-analysis showed reduced salivary vitamin C (SMD = −1.01; 95% CI: −1.381 to −0.64) and vitamin E levels (SMD = −1.33; 95% CI: −1.712 to −0.939), indicating weakened protection in both aqueous and lipid phases of the oral environment. Vitamin C is the major hydrophilic antioxidant capable of directly quenching the superoxide anion, hydroxyl radical, and singlet oxygen [147,148,149]. It serves as an electron donor to stabilize free radicals and prevent the initiation of oxidative reaction cascades, and it also regenerates oxidized vitamin E, thereby maintaining continuity of protection between aqueous and lipid phases [147,150]. Its biological importance extends beyond ROS neutralization, as it is a cofactor for prolyl and lysyl hydroxylases required for collagen synthesis and stability, which contributes to extracellular matrix integrity, proper epithelial anchorage, and barrier tightness [150,151]. Reduced salivary vitamin C levels in OL, reported by Kaur et al. (2015) and Rai et al. (2010), suggest the weakening of both antioxidant protection and structural mechanisms stabilizing the epithelium [38,42]. At the cellular level, vitamin C deficiency promotes the deregulation of cell–matrix interactions, increased epithelial permeability, and facilitated migration of inflammatory cells into the basal layer, potentially amplifying local cytokine production and ROS generation and sustaining chronic inflammation [151]. In addition, vitamin C modulates gene expression through effects on Fe^2+^- and α-ketoglutarate-dependent epigenetic enzymes such as dioxygenases involved in DNA and histone demethylation; therefore, deficiency may contribute to the epigenetic dysregulation of genes responsible for differentiation, cell cycle control, and stress responses, increasing susceptibility to malignant transformation [152,153,154].

In parallel, reduced salivary vitamin E levels in OL, described by Kaur et al. (2015) and Rai et al. (2010) [38,42], indicate weakened antioxidant protection in the lipid phase. Vitamin E is the principal lipophilic antioxidant of cellular membranes, where it limits lipid peroxidation by terminating free-radical chain reactions and stabilizing membrane structure [38,42]. Deficiency of vitamin E is associated with enhanced lipid peroxidation and the accumulation of reactive aldehydes, which can disrupt membrane-associated signaling and cellular homeostasis [155,156,157,158]. Lipid peroxidation products activate redox-dependent transcription factors, thereby promoting pro-inflammatory and pro-proliferative signaling [157]. In the context of oral leukoplakia, the reduced availability of vitamins C and E may increase epithelial vulnerability to oxidative stress, impair differentiation, and facilitate the accumulation of genetic and epigenetic damage, contributing to the maintenance of a premalignant phenotype [13,138,139,156,157].

Another key non-enzymatic defense component is uric acid (UA), which accounts for a substantial proportion of the total antioxidant capacity in plasma and saliva [157,158,159,160,161,162]. The meta-analysis demonstrated significantly reduced salivary UA levels in OL patients (SMD = −1.3; 95% CI: −1.805 to −0.786). Uric acid contributes to non-enzymatic antioxidant defense by directly scavenging ROS and chelating transition metal ions, thereby limiting hydroxyl radical formation [162,163,164,165]. Under chronic oxidative stress, increased consumption and limited regeneration of UA may lead to the depletion of local antioxidant reserves. Reduced salivary UA in oral leukoplakia may enhance metal-catalyzed lipid peroxidation and oxidative damage to DNA and proteins, sustaining a pro-oxidant microenvironment and processes linked to premalignant progression [13,165,166,167,168,169]. In this context, decreased UA should be interpreted primarily as a marker of weakened local antioxidant defense and increased susceptibility to free radical reactions [165,170].

Assessment of salivary redox balance in individuals with oral leukoplakia indicates complex relationships between clinical status and pathological alterations. Particular attention should be paid to total antioxidant capacity (TAC), for which significant alterations have been reported in patients with premalignant oral lesions, underscoring the relevance of global redox indices in oxidative stress evaluation [20,36]. Available evidence indicates that TAC, as a composite measure integrating multiple antioxidant system components, may change independently of individual antioxidant enzymes, suggesting the presence of compensatory adaptive mechanisms [28,51].

It has been hypothesized that increasing lesion surface area and clinical extent may be accompanied by rising salivary levels of immunological mediators, reflecting intensification of the local inflammatory response [171]. In parallel, changes in redox markers associated with the clinical and histopathological advancement of leukoplakia may indicate the progressive disruption of oxidative–reductive balance and the depletion of defense mechanisms [39]. Plasma-derived proteins, including albumin and haptoglobin, may contribute to these changes by entering the oral cavity under conditions of increased vascular permeability and enhanced gingival crevicular fluid exudation, thereby augmenting non-enzymatic antioxidant defenses through metal ion binding and radical scavenging [171,172,173,174].

Moreover, progressive inflammation is associated with increased ROS production by infiltrating neutrophils and damaged cells, leading to redox imbalance and activation or redistribution of antioxidant mechanisms in response to oxidative stress [133,175,176,177,178]. Such an influx of reducing components may paradoxically elevate global TAC without reflecting normalization of the biological activity of individual antioxidant enzymes such as SOD or CAT [179,180]. This dissociation supports the concept of the gradual exhaustion of specific defense systems as leukoplakia progresses [53].

Data suggest that in the advanced stages of mucosal disease, despite the high TAC, intensified oxidation of lipids and proteins occurs, supporting the link between histopathological advancement and redox homeostasis disturbances [20,28,39]. Several studies have attempted to relate the severity of these disturbances to clinico-pathological features of OL. Srivastava et al. (2019) reported a significant increase in lipid peroxidation markers (TBARS) and a decrease in enzymatic defense activity (GST) and UA with increasing epithelial dysplasia [34,39]. UA serves as a major non-enzymatic salivary antioxidant [162,181]. MDA is a biomarker of oxidative alterations, and GSH plays an important role in maintaining redox homeostasis, normal cellular function, and detoxification of carcinogens [39,182,183].

An important issue remains the impact of tobacco smoking, which is a potent source of ROS and directly modifies the redox biomarker profile by increasing pro-oxidant burden and inducing cascades that enhance oxidative stress, lipid peroxidation, and mitochondrial damage [184,185,186,187,188,189]. At the same time, smoking depletes antioxidant reserves regardless of the presence of any mucosal lesions, making it difficult to determine to what extent the redox disturbances observed in our meta-analysis are secondary to smoking itself (and other classical risk factors) versus reflecting an additional oxidative stress component independent of tobacco exposure and specifically related to the presence and progression of OL [20,185]. Thus, disentangling the etiology of the observed redox disturbances remains challenging.

To further explore potential sources of variability, subgroup analyses were performed for salivary MDA according to the analytical method used. Increased MDA levels in oral leukoplakia were observed consistently across assay-based subgroups, while substantial heterogeneity persisted within each subgroup, indicating that methodological differences alone do not explain the observed variability. In parallel, the overall certainty of evidence assessed using the GRADE framework was rated as very low, reflecting the observational design of the included studies, marked inconsistency, and limited precision. These considerations indicate that, although the direction of associations across biomarkers is largely consistent, the quantitative estimates should be interpreted cautiously and primarily as hypothesis-generating.

In summary, our findings suggest that OL is associated with a pro-oxidant shift in salivary redox balance and weakened defense mechanisms. This pattern includes enhanced lipid peroxidation (a significant increase in MDA and qualitative confirmation of increased TBARS) [33,34,39,67,68,70,76,77,190]. In parallel, deficits were observed in key antioxidant elements, including the glutathione axis (decreased GSH and GSH/GSSG), vitamins C and E, and uric acid, which may contribute to the persistence of a pro-oxidant mucosal microenvironment [156,157,162,165,191]. TAC-related findings suggest that with greater lesion extent, global antioxidant capacity may increase through compensatory mechanisms; however, this does not necessarily imply the normalization of specific enzymatic systems, nor effective protection against lipid and protein oxidation [174,175,176,177,178,179,180,181,182,183,184]. Overall, the salivary redox biomarker profile (increased MDA/TBARS and a trend toward increased 8-OHdG with decreased GSH, vitamins C/E, and UA) may reflect the biological activity of OL. Because saliva remains in direct contact with the lesion, it may have potential value for monitoring disease course and responses to interventions [130,131,132,149,150,151,152,170,191,192]. Notably, although most studies were rated as moderate-to-high quality (NOS 6–9), the weakest domain was inconsistent control of key confounders—particularly smoking/alcohol use and, in some studies, age and sex matching—which may have contributed to the observed heterogeneity.

### 4.1. Limitations

This systematic review and meta-analysis has several important limitations that should be considered when interpreting the findings. First, for several biomarkers, only a small number of eligible studies were available, which limited inferential power, reduced the robustness of pooled estimates, and precluded the reliable assessment of publication bias. Consequently, results for selected markers (e.g., TBARS, TAC, and 8-OHdG) should be regarded as exploratory rather than confirmatory.

Second, substantial to extreme heterogeneity was observed in most quantitative analyses, indicating pronounced clinical and methodological variability across studies. This heterogeneity could not be adequately explored through additional sensitivity or subgroup analyses, because detailed data on key effect modifiers—most notably smoking status, alcohol exposure, and other relevant participant characteristics—were inconsistently reported. As a result, the contribution of these factors to between-study variability could not be formally evaluated.

Third, in meta-analyses including only two independent studies, a fixed-effects model was applied a priori. This decision was driven by both methodological constraints and the limited ability of the statistical software to reliably estimate between-study variance (τ^2^) with such sparse data. Importantly, pooled estimates derived under these conditions should be interpreted with caution, as they primarily serve an exploratory purpose and do not provide robust evidence of a true underlying effect.

Fourth, lack of standardization in saliva collection protocols, pre-analytical handling, and analytical methods may have further compromised comparability between studies and contributed to the observed heterogeneity. In addition, control of confounding factors was inconsistent across studies, particularly with respect to smoking, which is known to substantially influence the salivary redox profile.

Fifth, the literature search was restricted to studies published in English, which introduces a potential risk of language bias and may have resulted in the omission of relevant data published in other languages.

Finally, the observational case–control design of all included studies limits causal inference. Nevertheless, despite the low overall certainty of the evidence, this meta-analysis was undertaken because no prior quantitative synthesis focusing specifically on salivary oxidative stress biomarkers in oral leukoplakia was available. Although the strength of inference remains limited, the generally consistent direction of effects across studies suggests potentially meaningful biological signals. These findings provide a rationale for further well-designed, adequately powered studies and highlight promising directions for future research rather than definitive clinical conclusions.

### 4.2. Future Directions

Because the evidence synthesized in this review and meta-analysis is largely exploratory, a key priority for future work is larger prospective studies with standardized saliva collection and analytical methodology, precise clinico-histopathological characterization of leukoplakia, and consistent control of the most important confounders. Such projects should also assess whether salivary redox biomarkers change in parallel with clinical lesion activity and can support the monitoring of treatment response.

From the perspective of clinical implications, the observed redox disturbance profile suggests that adjunctive therapies aimed at improving antioxidant status may be considered in OL management. Additionally, available data indicate that photodynamic therapy (PDT) used for OPMD treatment may influence the salivary redox profile. Therefore, it should be evaluated not only as a local treatment modality but also in the context of modulation of the oxidative microenvironment [193,194].

## 5. Conclusions

This systematic review and meta-analysis indicate that OL is associated with a salivary redox imbalance shifted towards a pro-oxidant state. Among the evaluated biomarkers, the increase in salivary MDA represents the most consistent and comparatively robust finding, supporting enhanced lipid peroxidation in OL. However, this conclusion is still based on a limited number of studies and is characterized by substantial between-study heterogeneity. In contrast, evidence for other oxidative damage and antioxidant markers—including 8-OHdG, GSH, UA, and the non-enzymatic antioxidants Vit. C and Vit. E—should be considered exploratory or hypothesis-generating, given the small number of available studies, extreme heterogeneity, and inconsistent control of confounding factors. Accordingly, all findings warrant cautious interpretation.

Although several pooled effects reached large standardized mean differences, such effect sizes reflect group-level separation rather than individual diagnostic performance and cannot be directly translated into clinical utility in the absence of validated cut-off values, sensitivity, and specificity estimates. Therefore, the observed magnitude of differences should be interpreted as biologically meaningful but not yet clinically actionable for diagnostic decision-making.

Further well-designed, prospective studies with standardized saliva sampling and analytical protocols are needed to validate salivary redox biomarkers and to clarify their potential role in monitoring disease course and therapeutic response, including antioxidant-based interventions and PDT.

## Figures and Tables

**Table 1:** Inclusion and exclusion criteria shown using the PECO framework.

Parameter	Inclusion Criteria	Exclusion Criteria
Population (P)	Patients of any age or gender	-
Exposure (E)	Presence of at least one OL lesion in the oral cavity	Studies not reporting OL lesions.
Comparison (C)	Systemically healthy controls without any OPMD’s lesion	Studies without healthy controls.
Outcomes (O)	Salivary oxidative stress and/or antioxidant biomarkers	Studies assessing only non-salivary samples (serum, plasma, tissue, gingival crevicular fluid); studies without oxidative stress or antioxidant outcomes; purely qualitative results or data insufficient for effect size calculation.
Study Design	Case–control or cross-sectional studies with both leukoplakia and control groups	Systematic reviews, narrative reviews, meta-analyses, case reports/series, conference abstracts, editorials, letters, theses; non-comparative designs; works not published in English; non-human or in vitro studies.

**Table 2:** Main characteristics of the case–control studies included in the review, presenting sample size, demographic data, assessed salivary redox biomarkers and methodological quality scores.

			Sample Size (M/F)		Age (Mean ± SD; Years)			
Author	Country	Study Design	OL	HC	OL	HC	Markers	Quality Score
Abdelkawy et al. (2020) [40]	Egypt	CC	20 (11/9)	20 (4/16)	47.6 ± 12.8	41 ± 7.7	MDA	8
Babiuch et al. (2019) [36]	Poland	CC	20 (11/9)	20 (9/11)	55.4 ± 10.52	52.1 ± 11.99	MDA, 8-OHdG, UA, GSH, GSSG, tGSH, GR, GPx, SOD, TAC	8
Guven et al. (2005) [41]	Turkey	CC	9 (6/3)	11 (ND)	42	34	MDA	5
Kaur et al. (2015) [38]	USA	CC	40 (20/20)	40 (20/20)	49 ± 5.9	48.9 ± 7	MDA, 8-OHdG, Vit. C, Vit. E	9
Metgud et al. (2014) [34]	India	CC	30 (ND)	30 (ND)	51.7	48.3	MDA, GSH	6
Rai et al. (2010) [42]	Belgium	CC	25 (13/12)	25 (ND)	ND	ND	MDA, 8-OHdG, Vit. C, Vit. E	7
Srivastava et al. (2019) [39]	Saudi Arabia	CC	40 (30/10)	40 (30/10)	45.2 ± 11.01	39.55 ± 9.11	TBARS, GST, UA	7
Shetty et al. (2013) [43]	India	CC	25 (ND)	25 (ND)	ND	ND	SOD	6

CC—Case–control study; HC—healthy control group; and ND—no data.

**Table 3:** Smoking status, epithelial dysplasia, and lesion site characteristics in oral leukoplakia patients across included studies.

Author	OL Group Characteristics	Smoking Status	Smoking Duration	Dysplasia				Lesion Site			
				No	Mild	Moderate	Severe	Buccal	Alveolar	Vestibular	Lingual
Abdelkawy et al. (2020) [40]	NPT NSD	ND	ND	ND				ND			
Babiuch et al. (2019) [36]	NPT NSD	NS = 4 CS = 11 FS = 5	ND	17	2	1	0	12	7	0	1
Guven et al. (2005) [41]	NPT	ND	>6 lat	9	0	0	0	5	0	0	4
Kaur et al. (2015) [38]	NPT NM	17.2 ± 3.2	ND	ND				ND			
Metgud et al. (2014) [34]	NPT NSD	ND	ND	0	13	9	8	12	8	5	5
Rai et al. (2010) [42]	NPT NSD	NS	NA	ND				ND			
Srivastava et al. (2019) [39]	NPT NSD	ND	20.8 ± 10.47	0	10	14	16	30	2	4	4
Shetty et al. (2013) [43]	NPT NSD	ND	ND	ND				ND			

NPT—no previous lesion treatment; NSD—no systemic diseases; NM—no malignancies in medical history; NS—non-smokers; CS—current smokers; FS—former smokers (smoked >100 cigarettes during lifetime but quit before sampling); ND—no data; and NA—not applicable.

**Table 4:** Saliva sampling and pre-analytical procedures used in the included studies.

Author	Saliva	Time	Sampling Method	Sample Volume	Sample Preparation
Abdelkawy et al. (2020) [40]	US	ND	Spitting	ND	Frozen to −20 °C
Babiuch et al. (2019) [36]	US	9:00–12:00 a.m.	Spitting	3 mL	Frozen to −80 °C
Guven et al. (2005) [41]	US	ND	ND	ND	Centrifuged at 4 °C for 10 min at 1200 rpm, then frozen at −20 °C
Kaur et al. (2015) [38]	US	ND	ND	ND	Centrifuged for 25 min at 3500 rpm, then frozen at −20 °C
Metgud et al. (2014) [34]	US	ND	Spitting	ND	ND
Rai et al. (2010) [42]	US	ND	Spitting	3 mL	Centrifuged at 4 °C for 5 min at 3000 rpm, then frozen at −80 °C
Srivastava et al. (2019) [39]	US	8:00–11:00 a.m.	Spitting	2 mL	Centrifuged at 4 °C for 10 min at 800 rpm
Shetty et al. (2013) [43]	US	ND	Spitting	ND	Centrifuged for 15 min at 10.000 rpm

US—unstimulated saliva, and ND—no data.

**Table 5:** Pooled results of the random/fixed effects meta-analyses for salivary redox biomarkers in patients with oral leukoplakia compared with healthy controls, including the number of contributing studies and participants, standardized mean differences with 95% confidence intervals, and heterogeneity statistics.

				Effect Size (OL vs. HC)			Heterogeneity			
Marker	*n* of Studies	*n* OL	*n* HC	SMD (95% Cl)	*Z*	*p*	Chi^2^	df	*p*	I^2^ (%)
MDA	6	144	146	1.47 [0.55, 2.39]	3.13	0.002	52.858	5.00	<0.001	91.13
8-OHdG	3	85	85	1.9 [−0.28, 4.08]	1.71	0.088	32.5	2	<0.001	96.92
GSH	2	50	50	−1.07 [−1.497, −0.645]	−4.93	<0.001	6.391	1	0.011	84.35
UA	2	60	60	−1.3 [−1.805, −0.786]	−4.99	<0.001	96.549	1	<0.001	98.96
Vitamin C	2	65	65	−1.01 [−1.381, −0.64]	−5.34	<0.001	7.981	1	0.005	87.47
Vitamin E	2	65	65	−1.33 [−1.712, −0.939]	−6.72	<0.001	9.034	1	0.003	88.93

**Table 6:** Subgroup analysis of salivary MDA according to assay methodology.

Subgroup		N	SMD [95% CI]	P-Heterogeneity	I^2^ (%)	*p*-Interaction
Assay method	TBARS	4	1.62 [0.34, 2.90]	<0.001	93.0	0.676
	ELISA	2	1.16 [−0.07, 2.39]	0.012	84.4	

**Table 7:** Leave-one-out sensitivity analysis for the meta-analysis of salivary MDA levels in oral leukoplakia versus healthy controls. Each row shows the pooled standardized mean difference (SMD) with 95% confidence interval and heterogeneity statistics (I^2^ and *p* value) after excluding the indicated study.

Deleted Article	I^2^ (%)	*p* Value	SMD [95% CI]
Babiuch et al. (2019) [36]	90.86	0.001	1.66 [0.65, 2.67]
Guven et al. (2005) [41]	91.65	0.001	1.66 [0.67, 2.65]
Kaur et al. (2015) [38]	66.43	<0.001	1.06 [0.55, 1.58]
Metgud et al. (2014) [34]	91.41	0.004	1.60 [0.52, 2.68]
Rai et al. (2010) [42]	92.36	0.010	1.44 [0.34, 2.54]
Abdelkawy et al. (2020) [40]	92.26	0.010	1.40 [0.34, 2.47]

**Table 8:** Direction of changes in salivary oxidative stress and antioxidant biomarkers in patients with oral leukoplakia compared with healthy controls, based on meta-analytic and qualitative evidence.

Biomarker	Direction of Change in OL vs. HC	Type of Evidence
MDA	Increased	Meta-analysis
8-OHdG	Trend toward increase	Meta-analysis
TBARS	Increased	Qualitative
GSH	Decreased	Meta-analysis
UA	Decreased	Meta-analysis
Vitamin C	Decreased	Meta-analysis
Vitamin E	Decreased	Meta-analysis
SOD	Inconsistent	Qualitative
TAC	Mixed/Increased with lesion size	Qualitative

## Data Availability

No new data were created or analyzed in this study. Data sharing is not applicable to this article.

## Supplementary Materials

The following supporting information can be downloaded at: https://www.mdpi.com/article/10.3390/antiox15020218/s1. Reference [195] is cited in the Supplementary Material.
