# Supplementary material for: Salivary Oxidative Stress and Antioxidant Markers in Oral Leukoplakia: A Systematic Review and Meta-Analysis
Source: Antioxidants (Basel). 2026 Feb 6;15(2):218. doi: 10.3390/antiox15020218 (PMC12938544; doi:10.3390/antiox15020218)
Supplement: Supplementary file 1 [file antioxidants-15-00218-s001.zip › Supplementary File S2.pdf]

Supplementary File S2 – GRADE Evidence Profile (Overall Body of Evidence)

This table summarizes the GRADE assessment for the overall body of evidence on salivary oxidative stress and antioxidant biomarkers in patients with oral leukoplakia compared with healthy controls. The GRADE approach was applied to the evidence as a whole rather than to individual biomarkers. Judgments were based on study design, Newcastle-Ottawa Scale (NOS) scores, pooled standardized mean differences (SMDs), heterogeneity statistics (I^2), confidence intervals, and consistency of findings across outcomes. Only studies included in the quantitative synthesis were considered.

GRADE Domain	Judgment	Rationale	Impact on Certainty
Risk of Bias	Serious	The evidence was derived exclusively from observational case-control studies. Although most studies were of moderate-to-high methodological quality according to the Newcastle-Ottawa Scale (scores 5-9), control of key confounders was inconsistent across studies, particularly smoking status, alcohol exposure, and demographic matching. Additionally, variability in saliva	Downgraded one level

		collection procedures and analytical methods may have introduced measurement bias.	
Inconsistency	Serious	Substantial to extreme heterogeneity was observed across most pooled analyses, including oxidative damage markers and antioxidant parameters (I^2 typically >80%). Although the direction of effects was generally consistent, the magnitude of effects varied markedly between studies. Sensitivity and subgroup analyses did not identify a dominant source of heterogeneity.	Downgraded one level
Indirectness	Not serious	All included studies directly evaluated salivary oxidative stress and antioxidant biomarkers in	No downgrade

		patients with clinically and/or histopathologically confirmed oral leukoplakia compared with systemically healthy controls, corresponding closely to the review question.	
Imprecision	Serious	Most outcomes were informed by a small number of studies with limited total sample sizes. Several pooled estimates were based on only two or three studies, and for some biomarkers confidence intervals were wide or crossed the line of no effect. No clinically established decision thresholds exist for salivary redox biomarkers in oral leukoplakia.	Downgraded one level
Publication Bias	Undetected	Formal assessment of publication bias was not feasible	No downgrade

because fewer than ten studies were available for each outcome. Publication bias cannot be excluded.

Overall Certainty of Evidence: VERY LOW
